# Editorial: Prognostic gene signatures in skin cancer

**DOI:** 10.3389/fonc.2023.1163642

**Published:** 2023-05-03

**Authors:** Gagan Chhabra, Wen-Qing Li, Colette Pameijer

**Affiliations:** ^1^ Department of Dermatology, University of Wisconsin-Madison, Madison, WI, United States; ^2^ Key Laboratory of Carcinogenesis and Translational Research (Ministry of Education/Beijing), Department of Cancer Epidemiology, Peking University Cancer Hospital & Institute, Beijing, China; ^3^ College of Medicine, The Pennsylvania State University, Hershey, PA, United States

**Keywords:** skin cancer, prognosis, gene signature, melanoma, editorial

Skin cancer is the most commonly occurring cancer worldwide with two major subcategories of melanoma and non-melanoma (1). Conventionally, skin cancers are treated with surgery and/or radiotherapy, however, if not diagnosed and treated early these malignancies can progress to locally advanced or metastatic stages. Over the past decade, a mechanistic understanding of immune regulation in skin cancer fueled the development of novel immunotherapy, including immune checkpoint inhibitors (ICIs), which has transformed the prognosis for many patients (2). Despite tremendous progress, currently, available therapeutics are still associated with sub-optimal responses due to drug resistance, especially against metastatic melanoma (3, 4). Hence, the identification of novel diagnostic, prognostic, and therapeutic targets is required for the management of skin cancers.

Bioinformatics analyses through several web servers and online tools (5) based on publicly available databases such as Genotype-Tissue Expression (GTEx) (6) and the Cancer Genome Atlas (TCGA) (7) have been used to identify potential prognostic markers in various cancers, leading to the establishment of predictive models to assess survival of individual patients (8). Importantly, prognostic gene signatures could help design novel strategies for the management of skin cancer. These studies are also important in guiding treatment selection and predicting patient outcomes. Moreover, the identification of potential biomarkers of skin cancers may also provide crucial information for the early detection of tumor relapse.

In this Research Topic, a total of 14 manuscripts were published focusing on prognostic genes in various skin cancer types including cutaneous melanoma (CM), uveal melanoma (UM), and cutaneous squamous cell carcinoma (cSCC). Overall, each team of investigators identified and validated either an individual gene or a multi-gene signature using several bioinformatics tools and/or *in vitro* experimental analyses. Below, we first discuss the studies focused on CM highlighting individual genes, and then a multi-gene signature, followed by the studies analyzing prognostic genes in UM and cSCC.

Zhang F et al. determined that eukaryotic translation initiation factor 6 (eIF6) may serve as a diagnostic and prognostic biomarker for predicting the survival of patients with cutaneous melanoma. Using immunohistochemistry (IHC) analysis of clinical specimens, the authors found that eIF6 was overexpressed in melanoma tumors compared to normal skin. eIF6 was also found to be significantly associated with decreased survival rates of patients with melanoma. Further, using *in vitro* experiments, this study showed that overexpression of eIF6 increased the proliferation and migration of melanoma cells. In addition, this study provided insights into the potential role of eIF6 in pan-cancer epigenetic regulation.

Zhong et al. described the oncogenic role of MYB proto-oncogene like 2 (MYBL2), a transcription factor that regulates the cell cycle. The authors showed overexpression of MYBL2 in malignant and metastatic melanoma patient samples, which was significantly associated with poor prognosis. The authors performed a loss-of-function study and demonstrated that MYBL2 depletion significantly decreased melanoma cell proliferation and migration as well as prevented cell cycle progression. They also showed that MYBL2 promoted the formation of melanoma stem-like cell populations, indicating its potential as a therapeutic target for treating resistant melanoma. Additionally, they constructed an MYBL2 regulatory network in melanoma by integrating RNA-seq and ChIP-seq data and identified three core target genes of MYBL2 that were EPPK1, PDE3A, and FCGR2A. Overall, this study concluded that MYBL2 may be a potential target for melanoma diagnosis and treatment.

Zhang J et al. showed decreased protocadherin 9 (PCDH9) expression in melanoma tissues compared to normal skin and pigmented nevus tissues using IHC analyses. The authors performed cell viability, cell cycle, apoptosis, and wound healing assay to determine the role of PCDH9 in melanoma. This study showed that an increase in PCDH9 could suppress melanoma cells and inhibit migration without exerting significant effects on the cell cycle. At a mechanistic level, the authors found that PCDH9 was negatively correlated with MMP2 and RAC1, while positively correlated with Cyclin D1. The authors concluded that PCDH9 could be useful as an independent prognostic marker for melanoma, and strategies to increase the expression of PCDH9 can be developed for the treatment of melanoma.

The study by Tong et al. aimed to identify new biomarkers for cutaneous melanoma and established a novel risk score system in melanoma prognosis. This study used univariate and multivariate Cox regression analyses to determine a model with four genes (ADAMDEC1, GNLY, HSPA13, and TRIM29). This four-gene risk score model was shown to be useful to predict the prognosis and treatment response in cutaneous melanoma. This model could be helpful to develop efficient therapeutic approaches against melanoma, however, additional studies are required to validate these findings.

Despite the success of immunotherapy that has transformed the prognosis for many cancer patients, no combined immune biomarkers are formally validated and recommended as a clinical tool for melanoma prognosis. In this regard, Zhang JA et al. described an immune-related prognostic gene signature in melanoma and correlated it with the immune infiltrating cells as well as the molecular subtypes of melanoma. The authors determined several differentially expressed genes such as IGHV1-18, CXCL11, LTF, and HLA-DQB1, which were associated with immune cell infiltration in patients with melanoma. In addition, the authors established a prognostic risk score for several types of immune infiltrating cells. These findings could be useful for future studies focusing on developing additional therapeutic strategies against melanoma.

Zhang H et al. constructed a 14-gene prognostic risk model based on cytolytic activity (CYT) level, an index of cancer immunity, in cutaneous melanoma using RNA sequencing data and clinical information from TCGA and GEO databases. The authors found that patients with high CYT levels had better prognoses. They also verified the expression of CYT-related genes in this prognostic risk model at the transcriptional as well as protein levels. In addition, the authors showed the utility of this model to predict and compare the response of patients to chemotherapy and immunotherapy. Altogether, this model could be helpful in the clinical management of melanoma.

Cutaneous melanoma is characterized by high immune cell infiltration in the tumor microenvironment (TME). However, an excess release of lactate, a major metabolic product, into the TME causes immunosuppression. Xie et al. determined the predictive value of lactate-related genes (LRGs) for prognosis and response to immunotherapy in patients with melanoma. This study found an inverse relation between the immune cells infiltration levels and clinical prognosis with patients’ risk scores based on the lactate-related prognostic signature and suggested that the low-risk cases could benefit better from immunotherapy. Overall, this lactate-related prognostic risk model may be explored in future clinical studies to predict survival and immunotherapy outcomes in patients with melanoma.

Interestingly, genes involved in DNA damage response could serve as promising candidates to predict response against ICIs. In this regard, Fischer et al. studied nine genes associated with xeroderma pigmentosum (XP), a genetic disorder caused by mutations in the genes of the nucleotide excision repair [7] pathway, which is primarily involved in the repair of ultraviolet radiation-induced DNA damage. As treatment with ICIs has been shown effective in XP patients with melanoma, the authors concluded that expression of XP-related genes could be used to predict melanoma prognosis as a well response to ICI treatment.

Zeng et al. established a prognostic nomogram based on metabolism-related genes (MRGs) and clinicopathological factors to predict melanoma prognosis. The authors identified several prognostic MRGs by comparing melanoma tumors with normal skin samples and suggested that two MRGs, tryptophanyl-tRNA Synthetase (WARS) and microsomal glutathione S-transferase 1 (MGST1) could be used as independent prognostic genes in melanoma.

Receptor tyrosine kinases (RTKs) are known to be overexpressed in tumors. In this regard, Lei et al. evaluated the association between overexpression of RTKs and survival in patients with melanoma based on several databases, which utilized IHC analyses. This study showed that overexpression of vascular endothelial growth factor receptor 2 (VEGFR2) was associated with worse patient survival in melanoma. Further, several other RTKs including epidermal growth factor receptor (EGFR), vascular endothelial growth factor receptor 1 (VEGFR1), insulin-like growth factor 1 receptor (IGF-1R), and mesenchymal-epithelial transition factor (MET) were also found to be associated with overall survival of patients with melanoma. This study concluded that overexpression of RTKs might be useful in accurate prognostic evaluation.

In another interesting study, Cheung et al. utilized next-generation sequencing (NGS) and performed hotspot mutation profiling on early-stage melanoma tumors obtained from patients at the Iowa City Veterans Affairs Medical Center. The authors found the highest prevalence of alterations in BRAF, TP53, NRAS, CDKN2A, KIT, and BAP1. In addition, they found significantly higher TP53 mutation in Veterans with prior history of melanoma. Overall, this study concluded that TP53 may be a useful marker to predict recurrent melanoma in the military population.

Luo et al. aimed to identify prognostic genes in uveal melanoma, the most common adult ocular tumor. The authors described prognostic implications of a ten-gene signature showing interactions with the immunodominant TME, which might be helpful to predict individual patient prognosis and develop new therapeutic strategies for patients with uveal melanoma.

Johnson et al. determined the role of complement factor H (CFH), a regulatory factor of the complement cascade, in the development of cSCC, the 2nd most common type of cancer in the US, following basal cell carcinoma. CFH has been shown to be associated with poor outcomes in different cancer types by affecting cell-mediated immunity. For this study, the authors utilized skin samples from sun-exposed normal individuals as well as cSCC patients. The results of this study showed that increased CFH levels in cSCC patients were independent of sun exposure and potentially linked to reduced effectiveness of the immune response leading to cSCC progression. The authors suggest that CFH levels might serve as a prognostic factor in cSCC.

Thind et al. performed whole-genome sequencing on lymph node metastases and blood DNA from cSCC patients with regional metastases of the head and neck. They designed a multifaceted computational analysis at the whole genome level to provide a deeper understanding of the genomic landscape of metastatic cSCC. The information provided in this study could be helpful to identify predictive biomarkers in primary as well as metastatic cSCC.

Taken together, the published studies in this Research Topic range from research articles to meta-analyses identifying various novel genes important in skin cancer prognosis and are appropriately collected under the title “Prognostic Gene Signatures in Skin Cancer”.

## Author contributions

GC wrote the draft, W-QL and CP edited and finalized it with substantial intellectual inputs. All the authors approve this editorial for publication.
